# *Trypanosoma evansi* and Surra: A Review and Perspectives on Transmission, Epidemiology and Control, Impact, and Zoonotic Aspects

**DOI:** 10.1155/2013/321237

**Published:** 2013-09-18

**Authors:** Marc Desquesnes, Alan Dargantes, De-Hua Lai, Zhao-Rong Lun, Philippe Holzmuller, Sathaporn Jittapalapong

**Affiliations:** ^1^CIRAD, UMR-InterTryp, 34398 Montpellier, France; ^2^Faculty of Veterinary Medicine, Kasetsart University, Chatuchak, Bangkok 10900, Thailand; ^3^College of Veterinary Medicine, Central Mindanao University, Mindanao, University Town, Musuan, Maramag, Philippines; ^4^Center for Parasitic Organisms, State Key Laboratory of Biocontrol, School of Life Sciences, Sun Yat-Sen University, Guangzhou 510275, China

## Abstract

This paper reviews the transmission modes of *Trypanosoma evansi*. Its worldwide distribution is attributed to mechanical transmission. While the role of tabanids is clear, we raise questions on the relative role of *Haematobia* sp. and the possible role of *Stomoxys* sp. in delayed transmission. A review of the available trypanocidal drugs and their efficacy in various host species is useful for understanding how they interact in disease epidemiology, which is complex. Although there are similarities with other mechanically transmitted trypanosomes, *T. evansi* has a more complex epidemiology due to the diversity of its hosts and vectors. The impact of clinical and subclinical disease is difficult to establish. A model was developed for buffaloes in the Philippines, which could be transferred to other places and livestock systems. Since *Trypanosoma evansi* was reported in humans, further research is required to investigate its zoonotic potential. Surra remains a potentially emerging disease that is a threat to Australia, Spain, and France. A number of questions about the disease have yet to be resolved. This brief review of the basic knowledge of *T. evansi* suggests that there is renewed interest in the parasite, which is spreading and has a major economic impact.

## 1. Introduction

Of all the pathogenic trypanosomes, *Trypanosoma evansi *has the widest host range and geographical distribution, worldwide. By comparison, its ancestor *Trypanosoma brucei* had a limited geographical distribution. This “evolution” is largely attributed to the new modes of transmission acquired by the parasite when it lost some of its genetic material allowing to implement the cyclical transmission in tsetse flies.

*Trypanosoma evansi* has a huge range of hosts receptive and susceptible to the infection, in which it exhibits highly variable clinical effects, depending on the host and the geographical area. These characteristics make surra not only a multispecies but also a polymorphic disease. In fact, it may even constitute a complex of diseases induced by a “group” of parasites named *Trypanosoma evansi* (or a group of sub species named *Trypanosoma brucei evansi*) [1]. In this review, we focus on the transmission of the parasite, its geographically variable epidemiology, the use of trypanocides to control infection, the difficulty of evaluating its impact, and lastly, the parasite's zoonotic potential. In conclusion, we recommend undertaking additional studies to further understanding of the disease epidemiology and dynamics in order to improve control. Every effort should be made to avoid the continuous geographical spread of the disease, including its circulation, emergence, and reemergence.

## 2. Transmission

*Trypanosoma evansi *may have multiple origins, geographical locations, hosts, and clinical features. In addition, it has multiple and complex means of transmission, which vary in terms of relative significance depending on the hosts and the geographical area. Indeed, *T. evansi* is transmitted in several ways, via biting insects, sucking insects, and vampire bats; transmission can also be vertical, horizontal, iatrogenic, and per-oral, with various epidemiological significances, depending on the season, the location, and host species. Similarly, leeches may transmit trypanosomes, and their potential for transmission of *T. evansi* should be explored, especially for buffalo leech (*Hirudinaria manillensis*) in Asia. 

### 2.1. Mechanical Transmission

Mechanical transmission by biting insects is the most important mode of transmission of *T. evansi* in camels, as well as in livestock and other large animals generally. People have suspected this to be the case for a long time: for example, in Algeria, *El debab* (means “fly”) or in India people thought that horseflies played a role in surra, known as “*makhi ki bimari*” (horsefly disease) in the Punjab region [2]. 

**Figure 1:**
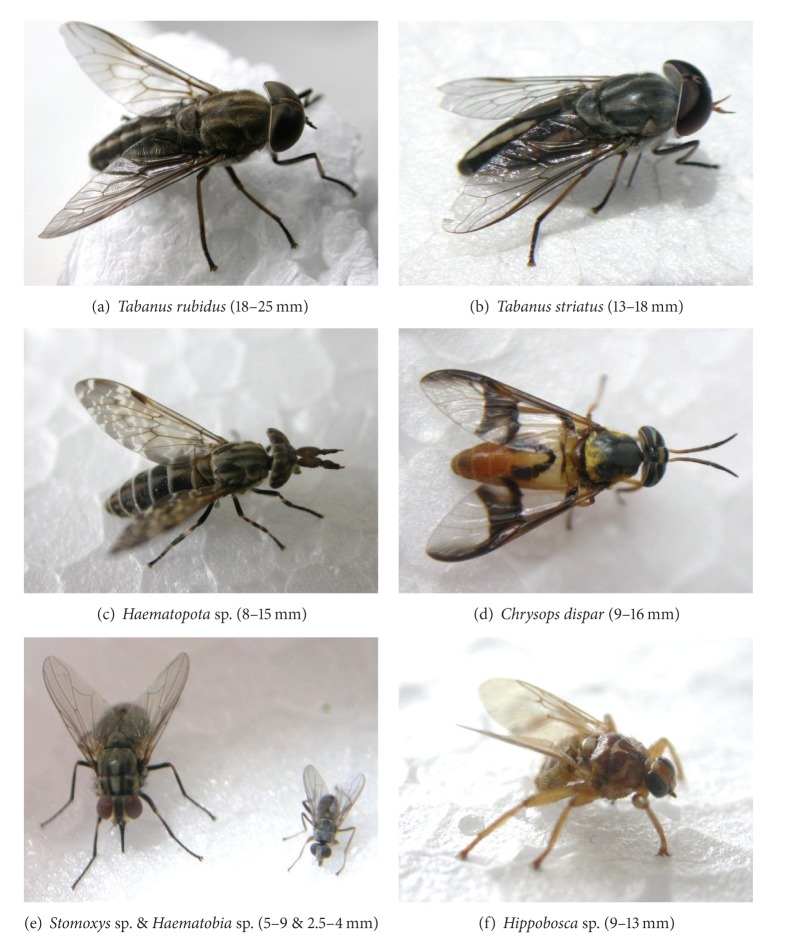
Some of the potential vectors of *Trypanosoma evansi* in Thailand.

Mechanical transmission is a nonspecific process, which can take place when a biting insect initiates a blood meal on an infected host, starts to feed on infected blood, is interrupted (by defensive movements of the host, e.g.), flies off from the infected host, and lands on another animal to begin its blood meal again. When the insect first attempts to feed on blood, its mouthparts can contain a small amount of blood via capillary strength, estimated at 1–12 nl in tabanids and 0.03 nl in *Stomoxys *[3]. The residual blood may be partially inoculated into another animal during the early stage of the next attempt to bite, when the insect inoculates a small amount of saliva (necessary for its anticoagulant properties) prior to sucking the blood of the second host [3–6]. A mathematical model has recently been developed; for cattle carrying a mean burden of 20–30 tabanids per head, the model indicated that the probability of transmission becomes significant when parasitaemia is above 10^6^ trypanosomes/mL [4]. Thus, in camels, which may exhibit very high parasitaemia (>10^8^ *T. evansi*/mL), tabanids and *Stomoxys* may be responsible for the transmission of *T. evansi*; possibly, *Haematobia* (Figure 1(e)) and hippobosques (Figure 1(f)) might act as well.

In biting insects, trypanosomes do not generally survive for very long. For example, their survival was estimated at 30 min with *T. vivax* in tabanids and even shorter in *Stomoxys* sp. [7]. Experimental research shows that the transmission is efficient when there is a short time lapse between two interrupted blood meals, that is, less than 30 minutes [8, 9]. Immediate mechanical transmission of this type can only occur in a group of animals (e.g., intraherd transmission). It leads to a high incidence of disease in a given herd. However, it may occur between herds of the same species (camels) or of different species (camels and goats, e.g.) at a water point. Transmission can also occur between wild and domestic herbivores, such as deer or capybaras when they graze with horses, cattle, or buffalo. This occurs in extensive breeding conditions in Brazil [10], for example.

An alternative to the immediate transmission of trypanosomes occurs when blood from the insect's gut or crop is regurgitated in the early stages of the blood feeding process. This could enable delayed transmission because parasites can survive in the stomach for 5–7 h in the case of *T. vivax* in tabanids [7]. Trypanosomes could survive for even longer periods in the crop of *Stomoxys* [11], where the absence of digestive secretion provides a more friendly environment. In experimental conditions of interrupted feeds, successful transmissions were obtained after 4 hours with *Tabanus nemocallosus*, 8 h with *T. rubidus* (Figure 1(a)), 24 h with *T. albimedius*, and up to 72 h with *T. striatus* (Figure 1(b)). The probability of success decreases drastically from 1 : 10 after 30 min to 1 : 1,000 after 6 hours [2]. However, tabanids are persistent feeders in natural conditions and, therefore, these results for delayed transmission would not apply. Once tabanids have initiated a blood meal, they make every attempt to complete it (even if it is interrupted), within a very short period of time; that is, they do not wait 4–72 h. Once satisfied, tabanids do not look for a host before 5–7 days have elapsed. *T. evansi *cannot survive that long; therefore, the probability of delayed transmission by tabanids is very low [6].

On the contrary, in early experiments with *Stomoxys*, it was shown that *T. evansi* could be transmitted 48 h after an infective blood meal [12]. This is unlikely to be due to residual blood on the mouthparts (survival was proven to be limited to 30 min in *Stomoxys*), but rather to the regurgitation of infected blood from the crop. However, it was demonstrated that *Stomoxys* may naturally have two blood meals in the same day or at 24-hour intervals [13]. This observation potentially has a very high epidemiological impact because a “split blood meal” would allow transmission over long time intervals. These intervals may range from a few hours to a few days. Thus, transmission may occur between herds in the same place (stationary insects) or between herds attacked by the same mobile insects. However, this experimental research needs to be confirmed. “Delayed mechanical transmission” might lead to the concept of “infective area,” such as a water point, where an infected herd could infect *Stomoxys* at a given time, which in turn would infect healthy animals (4–48 hours later), in the absence of contact between infected and uninfected herds.

Mechanical transmission of *T. evansi* is thought to be essentially due to tabanids and *Stomoxys*. However, Hippoboscids were previously suspected, especially in camels and horses (*Hippobosca equina* and *H. camelina*) [2]. Other insects, such as *Culicidae*, *Ceratopogonidae* may also have an important role in transmission in particular local conditions. Experimental transmission of *T. evansi *has been successful with *Aedes aegypti*, *Ae. Argenteus*, and* Anopheles fuliginosus*. However, the epidemiological significance has not been demonstrated (Kesler 1927 and Nieschulz 1928 quoted by Gill, [2]). It is not possible to establish an exhaustive list of the potential mechanical vectors of *T. evansi*. However, the most important are the largest and most abundant biting insects. The mathematic model of trypanosome transmission by tabanids developed by Desquesnes et al. [4] has shown that the incidence of transmission is directly linked to parasitaemia and the number of biting insects around the hosts. The transmission of the infection is related to a number of subparameters, including the size (and morphology) of the biting insect (volume of blood potentially transferred from one host to another) and the insect density. Thus, a high number of “small *Stomoxys*” can be as efficient as a low number of “large tabanids.”

**Figure 2:**
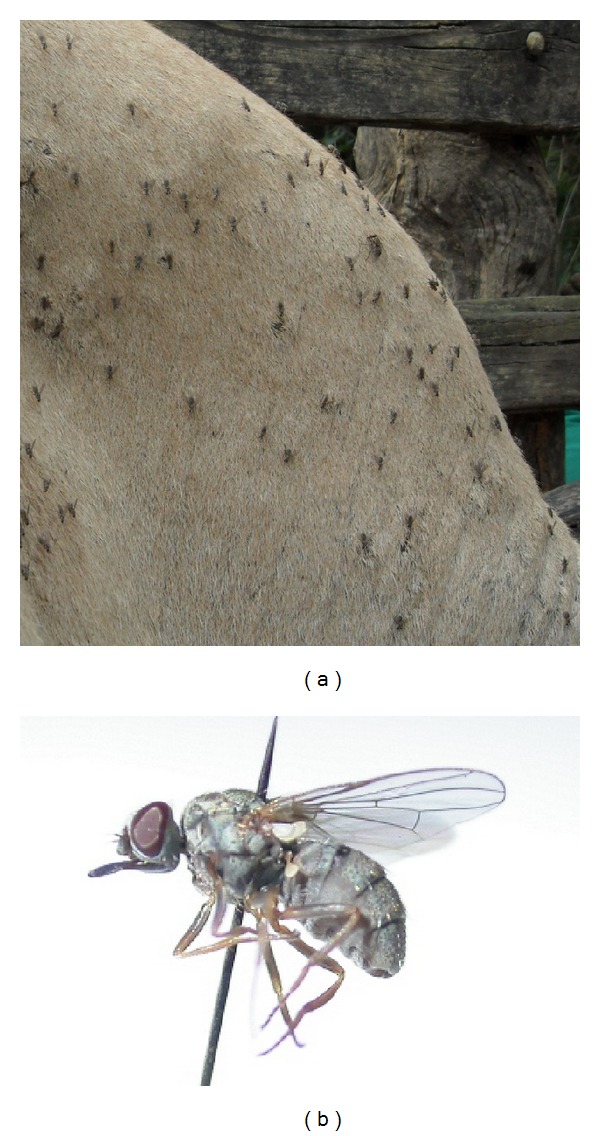
Potential role of *Haematobia* sp. (a) Common level of infestation by *Haematobia* sp. on the back of a cattle, Thailand (one *Stomoxys* sp. also visible); (b) *Haematobia* species (2.5–4 mm).

Gill [2] mentioned that successful experimental transmissions have been reported in no less than 29 *Tabanus* sp., including *Tabanus rubidus*, *T. ditaeniatus*, *T. immanis*, *T. rufiventris*, *T. malayensis*, *T. optatus*, *T. ceylonicus*, *T. partitus*, *T. striatus,* and *T. tenens*. He also reported several successful transmissions with *Haematopota* spp. (Figure 1(c)), *H. cingulata*, *H. truncate*, *H. irrorata*, *H. pungens*, *Chrysops dispar* (Figure 1(d)), *C. flaviventris*, *C. fasciata*, and even the lous *Haematopinus tuberculatus* (Mitzmain, 1913, quoted by Gill, [2]). *Lyperosia minuta* was also suspected on the basis of field observations, although experimental transmissions were unsuccessful [2]. Several demonstrations in experimental conditions were also reported for *Stomoxys* (Figure 1(e)) by Gill [2], with *Stomoxys calcitrans* and by other authors for *S. niger*, *S. varipes*, *S. taeniatus*, *S. pallidus*, and *Haematobosca squalida* [8, 9]. The role of *Haematobia* sp., the smallest of the Stomoxyine flies (2–4 mm), has never been demonstrated, probably due to its small size, which makes it inconvenient for laboratory experiments. However, it should be studied because the density of *Haematobia* observed in the field is frequently very high (Figures 1(e) and 2). In other words, their role may not be as negligible as their size!

Lastly, the potential role of reduviid bugs as mechanical vectors was demonstrated experimentally [14]. However, bugs do not move quickly from one host to another. Therefore, when they are infected when they first bite a host, they may not be able to move to another host in time to transmit the infection through another bite. Alternatively, infected bugs may be ingested by a host and, thus, transmit the parasite by the per-oral route. This occurs with *T*. *melophagium*, which is a stercorarian parasite transmitted to sheep when they chew the cyclical vector (*Melophagus ovinus*) [15].

Sucking flies can also transmit trypanosomes, via simple contamination of a wound, which may even be the feeding site of a biting insect. *Musca sorbens* was proven to transmit *Trypanosoma brucei rhodesiense* [16]. The same could also apply to *T. evansi* and was reported with *Musca crassirostris *[2]. 

Sucking flies may also increase the risk of mechanical transmission by other biting flies. In Thailand, some sucking flies on cattle have been observed escorting *Stomoxys* and pushing them away immediately in order to suck the blood at the biting site (M. Desquesnes, unpublished observation). By doing so, the sucking flies increase the risk of mechanical transmission by *Stomoxys* (by increasing the interrupted feeding). They also contaminate their own mouthparts for the potential transmission of a parasite to a neighbouring host. This phenomenon should be called “transfection” rather than “transmission.” 

### 2.2. Other Means of Transmission

Besides vector transmissions and the contamination of a wound, iatrogenic transmission caused by the use of nonsterile surgical instruments or needles may be of importance, especially during vaccination campaigns and mass treatments that could spread disease [17]. 

Sexual transmission or transmission from dam to calf or foal could occur in particular cases, when mucosae are altered or in cases of very close contact (licking) with parasitised secretions (mucus, lacrymation, etc.). This may be responsible for occasional direct horizontal transmission, although the real impact has not been estimated.

Transplacental infections have been described in *T. equiperdum* and *T. brucei* [18, 19]. Vertical transmission of *T. evansi* has also been demonstrated in several instances, as shown in a review on transplacental transmission of trypanosomes [20]. Indeed, in several cases of abortion in cattle and buffalo, the foetus was proven to be infected, directly or via mouse inoculation. Although vertical transmission may have a low impact on the incidence of the infection, it may be an important factor in the long-term maintenance of an infection in a herd or a given geographical area. It may also lead to the birth of healthy carriers that constitute a future reservoir for the multiplication of the parasite. Multiplication may occur under pressure from stress after a long period of subclinical infection. Therefore, vertical transmission may be the source of long-term resurgences.

*Trypanozoon*, especially *T. evansi*, may be transmitted by per-oral contamination. This mechanism could obviously occur quite easily when the oral mucosae are damaged. This may be frequent when carnivores eat infected prey. Dogs and cats living in the vicinity of slaughterhouses could be infected by eating fresh meat, blood, offal, or bones. Hunting dogs and wild carnivores could be contaminated in this way. The observation of circus tigers infected by *T. evansi *also suggests that infection is most probably due to eating infected meat [21]. Thus, the presence of *T. evansi* in French Guiana was revealed by the observation of a single case in a hunting dog [22], which demonstrates the importance of the role of the dog as a sentinel for surra [23].

Carnivores are not the only animals that are infected by the per-oral route. Under experimental conditions, it was demonstrated that the penetration of trypomastigote forms of the parasite can occur through the normal oral mucosae, in which *T. brucei* was able to multiply [24]. With *T. evansi*, it was shown that dogs and mice fed with meat and blood were infected [25], as well as rats fed on blood [26, 27]. However, the parasite is unable to survive in the stomach of carnivores and rodents because of the pH conditions. Therefore, in these hosts, the penetration of the parasite inevitably occurs through the oral mucosae. Conversely, the parasite can survive and pass through the oral mucosa, the oesophagus, and the stomach mucosa of vampire bats [28].

Other modes of transmission could be investigated, such as leeches, ticks (as carriers), and other blood feeders. 

### 2.3. Biological Vector: Vampire Bats

Transmission by the vampire bat is a new biological system that has been established in Latin America, thanks to the conquistadores who introduced both *T. evansi* and its hosts on the subcontinent [15]. Vampire bats are infected by the oral route when they leak blood from an infected prey (most often horses or cattle). As a host of *T. evansi*, bats may develop clinical symptoms and die during the initial phase of the disease (1 month). However, in the case of bats that survive, the parasite multiplies in the blood and is then found in the saliva of chronically infected bats or in bats that do not show any clinical symptoms. Later, infected bats can contaminate their congeners by biting, thus acting as true reservoir hosts. They can also contaminate livestock, acting as permanent vectors, capable of contaminating their host for a long period of time. Lastly, in the case of bats, the trypanosome may be transmitted from biter to bitten or vice versa [23]. Since the vampire bats can contaminate each other, a vampire colony can maintain *T. evansi* in the absence of the main host (horse), which makes them a true reservoir of the parasite. When feeding on horses or cattle, vampire bats are true vectors, in as much as they initiate infection that biting insects can then spread to other susceptible animals [15, 28, 29]. The vampire bat *Desmodus rotundus* acts as a host, reservoir, and biological vector of the parasite.

The different modes of transmission presented above may have variable importance depending on the host and epidemiological cycle. For example, biting insect transmission is very important in livestock or large animals more generally, vampire bat transmission is important in horses and cattle, though only in Latin America, and per-oral transmission is predominant in carnivores, vampire bats, and probably rodents. However, important data is missing in terms of our current knowledge, including data on the link between large animals and wild rodents, how rodents are contaminated, and the potential back infection from rodents to livestock or from carnivores to herbivores. This will be further discussed under the epidemiology section.

## 3. Control

Disease control is generally presented last, following the description of epidemiology. However, we decided to discuss disease control first because trypanocide use is now part of regular livestock management in all areas endemic for trypanosomoses. In other words, disease control has become part of disease epidemiology. Thus, it can only be understood if we take into consideration the continuous and cosmopolitan use of trypanocide drugs for livestock.

The control of a vectorial disease is classically divided into two sections: pathogen control and vector control. There are also various alternative means of controlling transmission, which can be combined as “means to prevent the infection.”

In the case of surra, in the absence of a vaccine against trypanosomes (due to a large repertoire of variable surface antigens), disease control is principally based on the use of trypanocides and preventive management methods to protect animals from infection.

### 3.1. Chemical Control of Parasites

As a blood parasite, *T. evansi* can be killed by injecting various trypanocidal drugs, providing that concentration of the chemical in the serum is lethal for the parasite. However, treatment might fail in the case of extravascular invasion or chemoresistance. 

Trypanocides can be divided into two categories. The “curative drugs” are used for treatment and have a short-term effect. They can kill the parasites, although they do not always eliminate 100% of them. The “curative/preventive drugs” are used for chemoprophylaxis. They not only kill parasites but also prevent any new infection or new circulation of parasites, due to the remanence of a sustainable curative dose in the serum of animals under chemoprophylaxis.

#### 3.1.1. Curative and Chemoprophylactic Drugs

Curative drugs aim to eliminate parasites from a sick animal. A drug could be regarded as “curative” when the dose used is able to eliminate all parasites. The most widely used curative trypanocide against surra is diminazene aceturate. However, other drugs can be used, such as isometamidium chloride (both curative and preventive), cymelarsan (so far, only recommended for curative treatment of camels), suramin, and quinapyramine (curative and/or preventive) [30].

Diminazene aceturate (DA) is an aromatic diamidine used to control babesia and trypanosome infection in ruminants. A curative dose of DA is administered via intramuscular injection to obtain a high concentration of the chemical in the circulating blood. The withdrawal period for the consumption of produce from cattle injected with DA is 21 days for meat and 3 days for milk [31]. However, the chemical dose in the serum actually suggests a longer withdrawal period of 30 and 21 days for meat and milk, respectively [32]. The dose recommended for the treatment of infections due to parasites belonging to the *Trypanozoon* subgenus is 7 mg/kg bodyweight (bw) of DA, via intramuscular injection. The reality in the field often reveals that a dose of 3.5 mg/kg bw is used to control surra. This could be for various reasons, including ignorance of the right dose or concern to save money by reducing the cost of treatment. Use of the “wrong” dose is based on the recommended dose for the treatment of infections by two other African *Trypanosoma* species: *T. vivax* and *T. congolense*. Diminazene aceturate is recommended in ruminants. Its use in horses and dogs is limited due to poor efficacy and tolerance in these species. Diminazene aceturate has been used for a long time. Consequently, trypanosomes have developed chemoresistance in most parts of the world [23, 31]. Using 3.5 mg/kg bw to control *T. evansi *can be considered as underdosing, as is often the case in Thailand and more generally in South East Asia. This dose can be regarded as a “premunition treatment,” when the host remains infected, (although clinically cured), contrary to the curative dose that eliminates all parasites. Such low dose treatment can lead to the selection of chemoresistant strains. In Thailand, for example, the inefficiency of DA in bovines, horses, pigs, and elephants has frequently been reported [33–36].

Isometamidium chloride (IMC) belongs to the phenanthridine family, as well as homidium chloride or bromide. However, the latter are highly toxic because they are DNA intercalating agents. Their mutagenic action was demonstrated early on [37, 38]. Therefore, their use in the field is not recommended. IMC is not known as a carcinogenic agent. It can be used for curative (0.5 mg/kg bw) and preventive (1 mg/kg bw) treatment of trypanosome infections in ruminants and horses, via intramuscular or subcutaneous injection. Alternate use of DA and IMC constitutes a “sanative pair,” which means that once resistance develops to one of the drugs, the other drug should be used to control the infection [30]. The withdrawal period for the consumption of produce in cattle injected with IMC is 23 days. However, it is obvious that the chemical can circulate in the blood for up to 4-5 months after injection [39]. Consequently, a safe withdrawal period should be much longer, from around 3 months (when 0.5 mg/kg is injected) to 6 months (when 1 mg/kg is injected). These withdrawal periods make IMC poorly adapted to beef or dairy cattle. Horses have a limited tolerance to IMC [40], although it remains an alternative to DA.

Melarsomine dihydrochloride (Cymelarsan) is the latest trypanocide to be developed. It was first available for commercial use in 1992. It is used to control surra in camels via deep intramuscular injection at a dose rate of 0.25 mg/kg bw [23]. Evaluations conducted on other host species suggest using rates of 0.25–0.5 mg/kg bw in horses, 0.5 mg/kg bw in cattle, and 0.75 mg/kg bw in buffaloes [41–43]. However, transient side effects (nervous signs) were observed in buffaloes treated with 0.75 mg/kg bw (Dargantes et al., unpublished data). Dogs have a satisfactory tolerance to the drug. It is recommended for the treatment of heartworm (*Dirofilaria immitis*) (Immiticide), at a dose of up to 2.5 mg/kg bw (via deep intralumbar injection). It can be used at a rate of 1-2 mg/kg bw against *T. evansi* infections. However, in the case of nervous infections in horses and dogs, even high doses, respectively, 0.5 mg/kg bw and 2 mg/kg bw, failed to cure the animals [44, 45].

Suramin is an ureic component which was used in horses and camels by intravenous injection. It was effective against *T. evansi* infection, although it is no longer used.

Quinapyramine belongs to the group of aminoquinaldine derivatives. Quinapyramine methyl-sulphate can be used to treat the infection by subcutaneous injection at a dose of 5 mg/kg bw. A more effective combination of quinapyramine sulphate and quinapyramine chloride (Triquin) can be used as a curative/preventive drug against *T. evansi* in horses and camels, administered by subcutaneous injection at a dose of 8 mg/kg bw. Local tolerance is sometimes low. However, the drug is quite efficient and the chemoprophylactic effect can last up to 4 months [46]. In cattle, the use of quinapyramine is not recommended because it may induce cross-resistance to both DA and IMC [47]. Its use should be restricted to horses and camels only.

#### 3.1.2. The Use of Trypanocides in Various Host Species

Buffalo, cattle, and small ruminants infected by *T. evansi* can be treated with DA (preferred drug) at a dose of 7 mg/kg bw by intramuscular injection. The withdrawal period for meat consumption should be >30 days. In the case of strong clinical signs, especially when parasitaemia is high, an initial injection of 3.5 mg/kg bw DA may be given to reduce the parasitaemia and a second injection of 7 mg/kg bw can be given 5 days later to ensure that all the parasites are killed. 

If the treatment is ineffective, the use of IMC is recommended at a dose of 0.5 mg/kg (withdrawal period for meat should be >90 days). Alternatively, the efficacy of melarsomine hydrochloride was recently demonstrated (no nervous signs were observed), at a dose of 0.5 mg/kg bw by deep intramuscular injection in cattle [45] and buffaloes (Dargantes et al., unpublished data). 

Horses, dogs, and cats can be treated with DA or IMC despite being quite sensitive to the drugs. It is essential to provide an adequate water supply to avoid a toxic effect on the kidneys, which can be fatal. Similarly, in the case of very high parasitaemia in cattle, half a dose of DA or IMC, followed by a normal dose 5 days later can be administered. Given that horses have a low tolerance to DA and IMC, the normal recommended dose can also be split into two subboosts (DA 2 × 3.5 mg/kg and IMC 2 × 0.25 mg/kg). However, the intervals between the subboosts should not be too long, otherwise the curative drug concentration in the plasma will not be reached. In such cases, injecting two subboosts with a 3–5-hour interval is recommended. DA treatment is not efficient in the case of nervous infection. Results of DA or IMC treatment may be satisfying, although chemoresistance is often observed, which limits the effectiveness of treatment. As an alternative, the efficacy of melarsomine dihydrochloride was evaluated in horses and dogs. The treatment can clear the parasite from the blood. However, in cases of nervous infection, it is inefficient and may cause death in the patients [44, 45]. Another alternative is the treatment of horses with quinapyramine sulphate and chloride (curative and chemoprophylactic effect), which provides durable protection to the animals. Nonetheless, we do not know whether the infected animals that receive such treatment are sterilised from the infection or whether they can carry the parasite in extravascular foci, such as joint fluids, cerebrospinal fluid, and aqueous humour of the eye, as has been demonstrated in camels [48]. However, if parasites do survive in an extravascular refuge and later attempt to reinvade the blood, they would be killed on reaching the blood given the chemoprophylactic drug's long-lasting action. In such conditions, keeping horses alive in enzootic areas might require regular treatment with the chemoprophylactic drug. Indeed, horse owners usually treat their animals regularly, both in Latin America and South East Asia [46]. In dogs, treatment with quinapyramine is poorly documented, although drugs are available on the market (Interquin). Another alternative treatment for dogs could be tried out, using 5–8 serial injections with DA at 3.5 mg/kg bw at a 2-3 week interval. In the absence of a trypanocide capable of establishing curative treatment for dogs, this type of strategy aims to enhance specific and protective immunity against the parasite. This premunition status can be expected within some months.

In camels, although a number of trypanocides have been used (DA, IMC, suramin, quinapyramine, etc.), melarsomine dihydrochloride is the ideal product (dose: 0.25 mg/kg bw), which can be increased up to 0.5 mg/kg bw if fully curative (sterilising) treatment is required for international trading. In enzootic areas, a dose of 3.5 mg/kg of DA can also be used. However, it can induce severe side effects and might not be sufficient to clear all parasites from the camel.

In pigs, little information is available on the control practices used for African trypanosomes. Quinapyramine may be used, as well as DA, though the latter appears to be of limited efficacy [36]. IMC and melarsomine dihydrochloride could also be used. However, experimental evaluations are necessary to validate the treatment protocols.

In Asian elephants, several attempts have been made with DA. Lower doses, such as 5 mg/kg bw, resulted in relapses [49, 50], while 8 mg/kg bw seems to be efficient [51]. Evaluation of melarsomine dihydrochloride at a dose of 0.2 mg/kg bw could also be evaluated (Frans Van Gool, personal communication).

#### 3.1.3. Strategies for the Use of Trypanocides

It is important to determine a strategy and the objectives of a treatment, whichever trypanocide is used. Indeed, in most of the highly enzootic situations, when the infection is not lethal, such as* T. evansi* in bovines, the treatment does not necessarily aim to completely eliminate the parasite from the animal. In practical terms, a “mild treatment” (3.5 mg/kg bw DA, e.g.) might be sufficient to kill the majority of parasites, ensure clinical improvement, and induce the release of a large amount of parasite antigens to enhance the host's immune response. Animals that are treated in this way, but remain infected, can cope with the infection because they develop an adapted immune response. This leads to the status of subclinical infection or healthy carrying. From the clinical point of view, farmers may think that such treatment is curative. Maintaining an efficient immune status is especially important for gestating animals in an enzootic situation. However, it is important to emphasise that low dose treatments potentially enhance the development of chemoresistance. 

In bovines, if the objective is to kill all parasites (to clear a farm from infection or prior to export, or for introducing an animal into a noninfected farm, etc.), a higher dose of DA or other chemicals should be used, such as 7 mg/kg DA bw (in the absence of chemoresistance), or 1 mg/kg bw IMC, or 0.5–0.75 mg/kg bw of melarsomine dihydrochloride. As previously mentioned, in dogs, a strategy of serial low doses of DA injections (3.5 mg/kg) could be attempted when sources of infection are out of control.

When the infection threat is lethal, such as *T. evansi* in horses and dogs, a different strategy is generally preferred, namely, to kill all parasites, as far as possible. The objective in this case is to achieve fully curative or “sterilizing” treatment, which requires the use of (i) curative drugs, such as DA 7 mg/kg bw (obviously with a high probability of treatment failure) or melarsomine dihydrochloride 0.5 mg/kg bw; or (ii) chemoprophylactic drugs, such as quinapyramine sulfate and chloride 8 mg/kg. However, in the case of an invasion of the nervous system, none of these drugs have yet been proven to be efficient. 

In horses, irrespective of the source of infection (from an inside extravascular focus in a carrier or from mechanical vectors that bring parasites from neighbouring infected hosts), the only option is to treat with quinapyramine sulfate and chloride at 8 mg/kg bw to protect the blood from parasite invasion. However, if the parasites reach the nervous system, the disease is always fatal. 

### 3.2. Preventing Infection

In addition to parasite prevention and control, vector control, or more generally, preventing infection is an important part of disease control, especially for highly susceptible species, such as horses and dogs. 

#### 3.2.1. Vector Control

In the case of tsetse-transmitted trypanosomes in Africa, vector control is quite effective at reducing the trypanosome pressure. The cyclical vectors can be specifically targeted using insecticide impregnated screens and insect sterilisation techniques can be used in a limited livestock breeding area [52]. 

Conversely, the control of mechanical vectors is not easy because of the diversity of tabanid species in a given area, their high mobility and prolificacy. In addition, the larval stages of tabanids are generally spread over a wide area and different species colonise various landscapes [53]. The ecological control of one species might help the development of another! Consequently, the ecological control of tabanids is not usually an option.

Tabanid control using insecticide sprays was proven to be efficient in small closed deforested areas in French Guyana [54]. However, even in this case, tabanid infestation reappeared 2-3 years after the end of the control campaign [23]. When tabanid control is carried out in an open area, it is not sustainable because tabanids move in from the surrounding areas to fill the ecological gap created by the control campaign. Implementation of tabanid control is rarely attempted because it is costly, unsatisfactory, unsustainable, and does not provide 100% cover from infection. Nonetheless, the control methods are described briefly below.

*Stomoxys* species differ from tabanids in that they develop within the livestock area or the farm and are closely related to the farming systems [53]. *Stomoxys* population control can be achieved through management methods (see description below). However, sustainable control using mechanical vectors is not possible because of their high mobility and prolificacy. Indeed, an adult female tabanid or *Stomoxys* may lay 100–200 eggs, 4-5 times in her lifetime. Therefore, total egg production ranges from 400 to 1000 eggs [53]. By comparison, a female tsetse fly produces one larva at a time, approximately 10 times, thus producing 10 flies in her lifetime. These are known as the “R” and “K” reproduction strategies, respectively [55].

Mechanical vectors use the “R” strategy. Their extreme prolificacy means that if only 2% of the eggs reach the adult stage, the tabanid population remains stable [53]. Hence, if more that 2% of eggs reach the adult stage, the population will increase. In order to control tabanid populations successfully, egg development must be kept below 2%.

**Figure 3:**
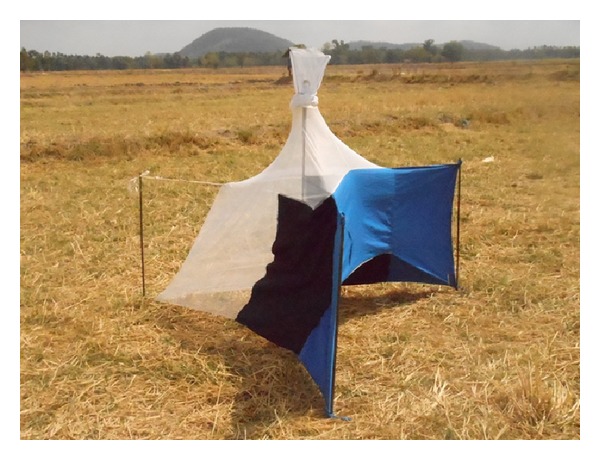
*Nzi* trap. A universal trap able to catch tsetse flies, tabanids, and *Stomoxys*, especially efficient for large size tabanids.

**Figure 4:**
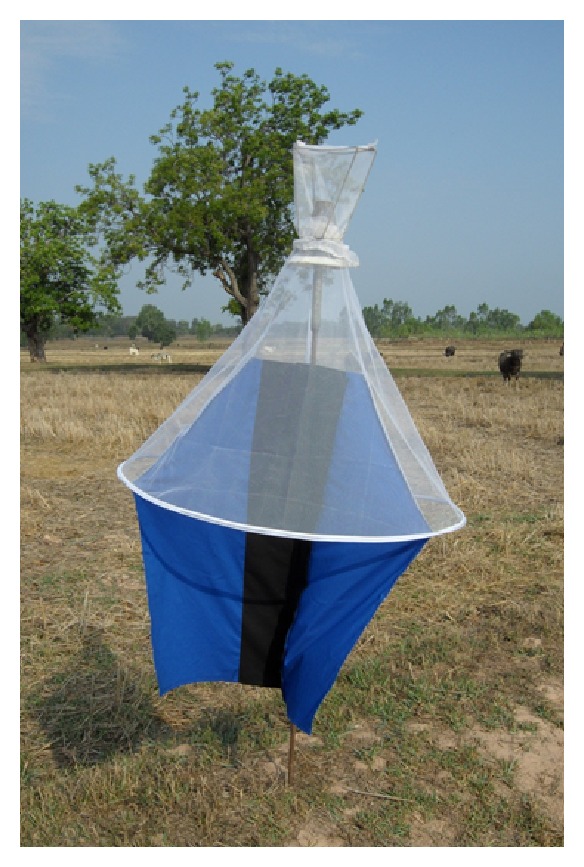
Vavoua trap. A trap designed for tsetse flies, especially efficient for *Chrysops* and *Stomoxys*.

**Figure 5:**
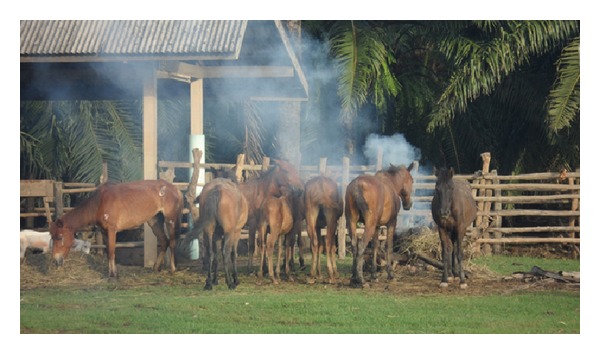
Smoke released to protect horses from biting flies, Surat Thani, Thailand.

**Figure 6:**
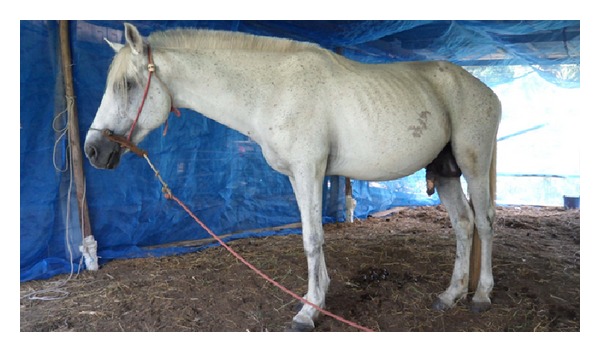
Mosquito net system on a stable to protect a horse against biting flies in an area of high infestation, Ratacha Buri, Thailand.

**Figure 7:**
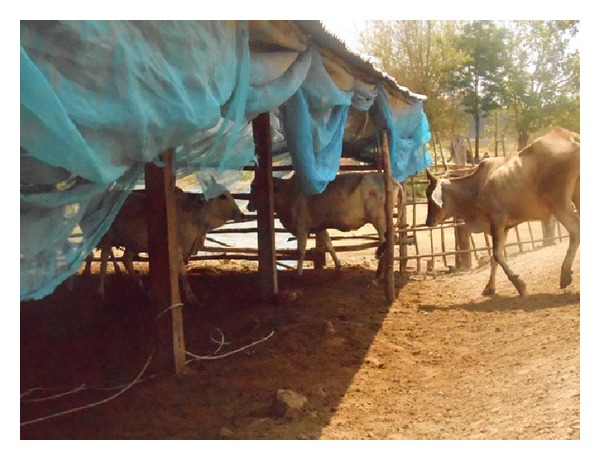
Fly proof system with mosquito net for cattle stable (Nakhon Sawan, Thailand).

The control of surra's vector populations can be attempted using traps and/or impregnated screens or using insecticides on livestock. The most efficient traps for mechanical vectors are the *Nzi* (Figure 3) and the *Vavoua* trap (Figure 4) [56, 57]. The *Nzi* trap can catch large tabanid species and *Stomoxys*, while the Vavoua trap catches small tabanid species, such as *Chrysops* (deer flies) and *Stomoxys* [58]. However, so far, these traps have been used to study insects and monitor control campaigns rather than for actual insect control. Spraying insecticides, such as deltamethrin on cattle, is efficient for controlling mechanical vectors [54, 59, 60]. However, the effect is relatively short-lived, which makes efficient control costly. The use of targets or impregnated screens, like those used in Africa against tsetse flies, was not evaluated for the control of mechanical vectors. It is an attractive alternative for the targeted control of biting insects. One of the traditional methods for controlling biting insects is the use of smoke released by slow fire (Figure 5). The smoke repels the insects. However, because it only covers a limited protected area, the animals in this area reduce their food intake [23]. Mosquito nets can be used to protect animals, though this is rare because of the expense. However, individual use does occur, such as for horses (Figure 6). It is possible to adapt fly-proof corals or stables for groups of animals, for example, cattle (Figure 7). Insecticide impregnation of mosquito nets is an alternative integrated method of control, which can help reduce biting insects/vector populations.

In Latin America, the vampire bat can act as vector, host, and reservoir of *T. evansi*. Consequently, vampire bat control is an integral part of surra control. The “Japanese net” can be used to catch vampire bats. It can also be used as a screen to protect livestock. In this case, it should be set up at night to create a screen between the bat colony refuge (forest area) and the livestock farm. Alternatively, the Japanese net can be used to catch a few bat specimens, which are then used to kill the colony. Captured animals are coated with drops of anticoagulant that contains an excipient, such as lanolin, before they are released. Once the animal has returned to the colony, it spreads the chemical to the whole colony by licking and contact. An anticoagulant, such as chlorophacinone, kills the bats within a few days [23, 61].

#### 3.2.2. Other Methods to Prevent Infection

In situations where it is difficult to control the biting insect populations, it may be easier to control transmission, though not with 100% efficacy. Tabanids are naturally persistent feeders [62] and they do not leave one animal to bite another if the latter is more than 50 metres away. Therefore, 200 m is considered to be a safe distance for mechanical transmission by biting insects [62–64].

However, separating bovines from equines is highly recommended to avoid the transmission of *T. evansi* from a buffalo or cattle reservoir to highly sensitive horses. To avoid any risk of transmission (even that of occasional contact with animals that have escaped), it is advisable to breed cattle and horses in completely different areas that are at least several kilometres apart.

The case of carnivores is quite unusual. Carnivores may be infected when they eat the bones, flesh, or blood of an infected animal that has only just died. Rodents, which are omnivorous, may become infected like carnivores. *T. evansi* can be transmitted via oral infection as demonstrated in a trial in which per-oral blood was given to rats and mice [26, 27]. To avoid such infections, the dead animals' carcasses should be eliminated as soon as possible and dogs, especially stray dogs, should be contained around slaughterhouses, as well as on livestock farms in general.

In addition to per-oral contamination, dogs may also contract the infection from biting flies, especially the dog fly, *Stomoxys*, when they live in the vicinity of reservoir animals, such as cattle and horses. As mentioned above, host species must be well separated to avoid the interspecific host circulation of parasites.

#### 3.2.3. Preventing Introduction into a Noninfected Area

As was recently observed in Spain and France [65], healthy or inapparent carriers may be responsible for introducing the parasite from infected to noninfected areas. A number of measures, that are currently being studied, could be applied in order to avoid this type of introduction. The detection of carriers is based on laboratory detection according to the guide of methods recommended by the World Organisation for Animal Health (WOAH) in its terrestrial manual, Chapter 2.1.17. It is available online at the following address:

(http://www.oie.int/fileadmin/Home/fr/Health_standards/tahm/2.01.17_TRYPANO_SURRA.pdf). 

Diagnosis techniques for surra are based on four types of examination (referred to below as “surra tests”): microscopic examination, DNA detection by PCR, CATT/*T. evansi* and ELISA *T. evansi* (detailed protocols are available at the above link).

For the international trading of animals, the following guidelines could help avoid the introduction of infected animals into noninfected areas.

- Two quarantines should be applied for the international trade of equines and/or camelids (which could be extended to any mammal) from an infected country to a noninfected country: a 4-week quarantine at the exporting farm and a 4-week quarantine at the importing farm.
- To qualify for trading, an animal should originate from a noninfected farm in a nonsuspect area, and be negative to surra  tests twice at a 3-4 week interval during each of the quarantines.
- A farm is considered to be in a nonsuspect area if there have been no reports of surra in the previous 3 years within a 30 km radius of the farm.
- A noninfected farm is a farm located in a nonsuspect area, which only permits the introduction of animals that are negative to the surra tests and that originate from noninfected farms located in a nonsuspect area. To obtain the status of noninfected farm, all mammal species on the farm must be negative to surra tests twice at a 3-month interval. To maintain the status of noninfected farm, all mammal species on the farm must be negative to surra tests when tested every 10–12 months.

If these measures were adopted, they would considerably help to control the circulation of animals infected with *T. evansi*.

## 4. Epidemiology

Surra is a disease that can show the following: (i) various symptoms in a given host (from subclinical evolution to abortion or death, with or without vascular, nervous, or genital signs); (ii) various symptoms from one host to another (mostly lethal in horses, acute or chronic in camels, variable in bovines and buffaloes, acute in dogs, and generally mild but sometimes acute in pigs, sheep, and goats, etc.); as well as (iii) various aspects in different places (surra in buffaloes and cattle is virtually absent in Latin American, although it is a major constraint in South East Asia). The epidemiology of a disease depends on the characteristics of a pathogen, its hosts, reservoir, and vectors and their environment and interrelations. Consequently, in the peculiar case of surra, which is a multispecies disease, it can exhibit highly variable characteristics because of its highly complex epidemiology.

The study of the epidemiology of surra requires various specific diagnostic tools. Detailed procedures are available from the World Animal Health Organisation (WAHO/OIE) website, terrestrial manual, under Chapter 2.1.17 *Trypanosoma evansi* infection (surra), as indicated above. For this reason, we only present a summary of the techniques and their characteristics, as required for a comprehensive description of surra's epidemiology.

### 4.1. Diagnostic Tools

As described above, clinical signs are only indicative of surra. The definitive diagnosis involves laboratory analysis, either by using parasitological or molecular tools to demonstrate the presence of the infection or by using serological tools to prove immune contact.

Parasitological examinations are usually conducted using blood, although other biological materials can be used, such as cerebrospinal fluid (in the case on nervous signs), joint fluid, or lymph node fluid. Microscopic observation (×400–500) of fresh blood is easy to carry out. However, it is of limited sensitivity because it detects parasites when parasitaemia is above 10^5^ trypanosomes/mL of blood. Enrichment methods are widely used, namely, Hematocrit Centrifuge Technique (HCT) [66] or dark ground Buffy Coat Method (BCM) [67]. They increase the sensitivity of the test down to 100–200 trypanosomes/mL. If high sensitivity is required, inoculating laboratory rodents can reveal infection. It lowers the minimum level of parasitaemia detected to 20–50 parasites/mL.

In addition, molecular evidence of *T. evansi* DNA can be tested using PCR with a number of primers specific for the subgenus *Trypanozoon*, or to species levels [68]. Despite being relatively expensive and technical, PCR is generally used to improve the sensitivity of the detection. Comparative studies have led to the recommendation of TBR primers [69] as the most sensitive primers for detecting *T. evansi* [70], and the Phenol-Chloroform method [71] as the most sensitive DNA preparation method [72]. A combination of these methods provided a sensitivity of around 5–10 trypanosomes/mL of blood (or other fluid).

In addition to the use of parasitological or molecular tools for detecting *T. evansi* infection, serological tests that prove the immune contact between the host and the parasite are quite useful. They can be applied to investigations at herd or population level (prevalence or incidence studies), follow-up (seasonal or interannual variations), or control method assessment (trypanocide treatment or vector control). The most common tools are the Card Agglutination Test for *T. evansi* (CATT/*T. evansi*) [73] and the ELISA *T. evansi* [74, 75]. CATT can detect immunoglobulin M and, therefore, early infections, whereas ELISA is generally used to detect immunoglobulin G, that is, established infections. Consequently, these tests are complementary and work well together. ELISA *T. evansi* is quite robust, regardless of the host species. It provides the same range of sensitivity and specificity (90–95%) in the various host species investigated, for example, camels, cattle, buffalo, and horses. The sensitivity of CATT *T. evansi* varies from one host to another. CATT seems highly sensitive in camels and horses, although it has a very low sensitivity in cattle (12%), even under experimental conditions [41].

### 4.2. Africa and the Middle East

In Africa and the Middle East, *T. evansi* is responsible for an acute or chronic disease principally found in camels, horses, and dogs in the north of the tsetse belt [76]. In camels kept close to the tsetse belt, some cases of *T. brucei brucei *have been recorded. *T. congolense* infections are fatal to camels. Therefore, camels should not be allowed to enter the tsetse belt unless they are permanently protected with the use of chemicals. Consequently, *T. evansi* is not found in the tsetse belt. Thus, the epidemiology of surra in Africa is mainly governed by camel infections. The latter are seasonal because the vectors' activity is seasonal and the disease is expressed seasonally, at times when animals are exposed to stress from overwork, food shortages, and/or insufficient or poor quality water [77]. For example, in Mauritania, by using CATT and IFAT blood smears, it was shown that *T. evansi* infection was widespread in the country, with an overall prevalence of 1.3% by parasitological detection. This level reached 18.4% to 31% with serological tests [78]. Other host species, such as goats, may be infected occasionally and could act as a reservoir. However, their impact was never demonstrated [79]. In cattle, especially transhumant herds, that spend part of the year within the tsetse belt and the rest of the year in the northern region, it is difficult to distinguish between infections due to *T. brucei* and *T. evansi*. The latter is probably rare because, even under experimental conditions, the infection of African cattle by *T. evansi* proved to be difficult as a consequence of their low susceptibility [80]. The transmission of *T. evansi* can only occur if the “donor host” exhibits high parasitaemia. This is because *T. evansi* is mechanically transmitted by biting insects (due to the very small amount of blood transferred from one host to another). In Africa, only camels and horses may be a source for this type of transmission. Hosts that have a low susceptibility, such as cattle and goats, are likely to constitute dead ends, even if they may occasionally be infected when close to infected camels or horses. Finally, given that camels and horse cannot enter the tsetse belt without being at risk from Nagana, which is fatal to both hosts, and because other hosts that could be infected by *T. evansi* do not exhibit sufficient parasitaemia to play an important role in surra's epidemiology, there is a reciprocal exclusion of Nagana in the southern territory (among tsetse flies, livestock, and wild animals) and surra, which is restricted to the northern region (among mechanical vectors and camels).

In the Middle East and towards Asia, the geographical distribution of *T. evansi* is closely related to that of camels and dromedaries [15]. However, no difference was observed in terms of the pathogenic effects of the parasite in this host species, which like horses are highly sensitive to the infection.

Overall, surra affects mainly camels with acute and chronic infections that cause death. Infection is contracted during the rainy season when there is a peak level of biting insects. Camels constitute the main reservoir of *T. evansi* in this region.

### 4.3. Latin America

In Latin America, the disease is called *Mal de Caderas* (Brazil), *Murrina* (Central America), or *Derrengadera* (Venezuela) (Wells 1989). *T. evansi* is principally pathogenic in horses and induces outbreaks with very high morbidity and mortality. It also affects buffaloes (*Bubalus bubalis*). In Venezuela, although infection in buffaloes by *T. evansi* showed significant signs such as spleen, liver, and glandular enlargement, together with lymphoproliferation, the economic impact of infections has not been assessed [81]. *Trypanosoma evansi* regularly affects dogs (especially hunting dogs) and even cats. In both cases, the disease is usually fatal. In Latin American cattle, sheep, goats, and pigs, *T. evansi* is generally considered as a low pathogenic agent. It is regularly found in a wide range of wild reservoirs, including capybaras (*Hydrochoerus hydrochaeris*), which is the most well known, together with white tail deer (*Odocoileus virginianus chiriquensis*), brocket deer (*Mazama satorii*), coati (*Nasua nasua*), vampire bats (*Desmodus rotundus*), wild pigs (*Tayassu tajacu*), Guinea pig (*Cavia porcellus*), wild dog (*Canis azarae*), and ocelot (*Felis pardalis*). Llamas are also receptive to the disease and infected animals have been found, although little is known about its impact [23]. There is no obvious link between wild and domestic fauna. In some places, the prevalence of infection may be very high in capybara and coati, while it remains low in horses [82]. In French Guyana, the parasite has never been found in livestock, including horses, but it was first described in 1995 in a hunting dog, which was probably infected by wild fauna when hunting in the forest [23]. Surra remains a major disease in Latin America, especially because horses (sensitive host) are used for herding cattle (reservoir) in extensive conditions in Venezuela and Brazil, for example.

Overall in Latin America, surra is predominantly a disease that affects horses. However, a large range of wild and domestic mammals can act as a reservoir. In most cases, farmers use chemoprophylactic drugs regularly to protect horses against *T. evansi* (isometamidium or quinapyramine). This treatment ensures that they stay alive and efficient for work. As a result, two groups of livestock are kept in close contact: a low susceptible reservoir made up of bovines and a highly susceptible host made up of horses under chemoprophylaxis.

### 4.4. Asia

In Asia, the geographical distribution of *T. evansi* is spreading steadily. It is present in large areas in India, China, and Russia [83, 84]. It is sometimes difficult to distinguish it from *T. equiperdum* [85]. It is present in* Camelus bactrianus* and horses in Mongolia, with low prevalence. It is more frequent in Uzbekistan and Kazakhstan. In South East Asia, it affects principally horses, dogs, and buffaloes (*Bubalus bubalis*), as well as cattle, pigs, and deer. It has been described in tigers in India [21], as well as in Thai elephants [86].

In the water buffalo, *T. evansi *causes production losses, abortion, and early calf mortality. It also has immuno-suppressing effects, which decrease the efficacy of some vaccines (especially of the vaccine for hemorrhagic septicaemia). In bovines, its pathogenicity in Asia is superior to that of African and American strains. We do not know whether the difference is due to the presence of more sensitive dairy breeds in Asia, or if the local populations of *T. evansi* are more pathogenic to cattle, or both.

In India, surra is present all over the country in various hosts, such as cattle, buffaloes, camels, donkeys, dogs, and horses [87]. A recent survey carried out on horses showed a maximum seroprevalence (20%) for *T. evansi* infection in Uttar Pradesh. There was an overall seroprevalence of 11% in north and north-western regions of India, which confirmed that surra is endemic in equids in these areas [88].

In Thailand, a study on seroprevalence carried out in dairy cattle demonstrated the presence of the parasite in most parts of the country. The mean seroprevalence was 8%, ranging from 0 to 100% at farm level and 25% of dairy cattle are exposed to the infection [89]. Similar studies conducted on buffaloes and beef cattle showed seroprevalence of 10–12% (Desquesnes, unpublished data). Molecular evidence of *T. evansi* was also obtained in various wild rodents [90, 91]. However, their role in the epidemiology of the disease is not known. In horses, several outbreaks are recorded every year and are frequently fatal. Indeed, serological studies show very low evidence of positive animals; in other words, there are few survivors after the outbreaks [44, 92]. Elephants are affected by surra. Cases are reported rarely but regularly. They may be fatal or develop into a chronic or subclinical evolution, depending on the case [50]. Surra outbreaks occur seasonally and are generally linked to the activity of biting flies. In Northeast Thailand, seasonal occurrence is observed at the beginning of the rainy season (June-July) and in winter (October-November) [93]. Bovines (cattle and buffaloes) exhibit moderate signs and impact. However, they constitute a permanent threat to themselves and horses, which may die or survive under permanent chemoprophylaxis.

In the Philippines, over the past decade, the number and severity of surra outbreaks have increased dramatically. The highest mortality is in horses, carabao (Asian water buffalo), and cattle. As a result, the Philippine government now regards surra as the second most important livestock disease [94]. Indeed, surra has emerged as the most important cause of livestock mortality in the Philippines, prompting the government to implement a national control strategy.

In Indonesia, the disease appears in sporadic outbreaks, mainly in horses, buffaloes, cattle, and dogs, although it is also present in sheep, goats, pigs, and wild animals [94–96]. The parasite was found throughout most of the archipelago. Its regular occurrence suggests the existence of enzootic stability, including an efficient reservoir [95, 96]. However, an update of information is required.

In Vietnam, Laos, and Cambodia, the disease occurred especially in horses, buffaloes, and cattle, although it was given little attention. Serological surveys demonstrated the presence of infection in all the areas investigated. Limited means are available for carrying out studies on surra since priority is given to other diseases in these countries. Hence, the situation is not well documented.

Similarly in Malaysia, although the disease has been known for years, a national survey has not yet been organised to evaluate its impact. Surra is regularly detected in horses, deer, pigs, buffaloes, cattle and, rarely, in dogs. It was also reported in Sumatran rhinoceroses [97]. In Malaysia, a seroprevalence survey carried out in 2012, for dourine in horses using CFT did not reveal its presence [98]. Diagnosis is routinely carried out using a mouse inoculation test and buffy coat examination. Thin blood smears are also conducted in regional laboratories. Prophylactic treatment is administered to livestock in high-risk areas where cattle and buffaloes live in close proximity to pigs or horses [99, 100]. In domesticated deer, the infection is observed regularly. Fulminating parasitaemia is detected when the animals become weak and recumbent. Nervous symptoms are not clearly evident; however, fatality is most often observed during the outbreaks.

*Trypanosoma evansi* is not present in Australia, but it may spread eastward from Indonesia to Papua New Guinea and then Australia [101].

Overall in Asia, surra is mainly a disease of horses and buffaloes. It benefits from a large reservoir made up of buffaloes, cattle, deer, and possibly wild animals, such as deer and rodents. On cattle farms, little attention is given to the disease, even though it may cause serious economic losses, via abortion, weight loss, and immunosuppressive effects. An evaluation of the economic impact is needed to determine whether it would be profitable to eliminate the infection. Horse breeders generally avoid close contact between buffaloes and horses to avoid the risk of infection, which is generally fatal and uncontrollable because of the limited efficacy of trypanocides [34, 36]. When horses are bred in the same area as cattle or buffaloes, farmers regularly use chemoprophylactic drugs to protect horses against *T. evansi* (isometamidium or quinapyramine). Nonetheless, *T. evansi* remains a permanent threat to livestock throughout South-East Asia, with a decreasing gradient of impact for horses, buffaloes, dogs, cattle, deer, pigs, sheep, and goats.

## 5. Impact

There is limited information on the impact of surra among livestock in endemic countries, particularly (i) its impact on host population dynamics and demographics, (ii) the economic losses due to the disease, and (iii) social impact on animal owners. It is common knowledge that surra is an economically important disease, which causes high mortality, low milk and meat production, poor carcass quality, reduced reproductive performance, decreased draught power and manure production, and immunosuppression in livestock [94, 102–105]. Yet, only few studies have quantified the economic value of the losses (including expenditure on diagnosis, treatment, and replacement of lost animals) for limited animal species and for limited locations. Little information is available on the financial benefits of treating/controlling surra in infected animal populations.

The impact of surra on host population dynamics and reproduction has been extensively investigated for buffaloes in the southern Philippines [106]. Surra has a significant negative impact on buffalo populations, causing high mortality and reproductive losses. In particular, in surra endemic areas, buffalo herds have fewer calves, 50% lower calving rate and higher removal rates (including adult mortality and early calf deaths), than buffaloes in areas where surra is not detected. Higher mortality has been recorded amongst young buffalo cows aged 2–8 years old in surra-endemic areas compared to surra-free villages (9.1% versus 0.1% mortality, resp.). Given the decrease in the buffalo population in surra-endemic areas, replacement buffaloes are regularly imported because they provide essential draught power for farm operations [106, 107]. This impact may also be true for other animal species that are susceptible to *T. evansi*. However, further investigations are required to validate the impact of surra on other hosts.

The low calving performance among buffaloes in Mindanao (in the Philippines) is closely linked to abortion and infertility [107]. In Thailand, abortions and reproductive failure due to surra have also been demonstrated in buffaloes [108], cattle [104, 109, 110], camels [111], and horses [34, 112]. The death of buffalo cows during their most productive phase reduces their life expectancy (by almost half) and has a major impact on farmers. Females at this age are highly valued for draught power and as breeding animals for replacement or sale (to provide additional income) or home consumption. Surra has been proven to cause mortality in buffalo after experimental [113] or natural infection [83, 102, 114, 115]. Whilst mortalities in draught buffalo caused by surra could be partly associated with stress due to overwork, other factors such as malnutrition, concurrent infections, and adverse climatic conditions may contribute to the animals' reduced resistance and higher susceptibility to the disease [102, 115]. Surra is also lethal in other livestock species, such as horses [116–118], camels [111, 119], guanaco [120], cattle [86, 121], goats [122–124], sheep [125], and even pigs [126]. Indeed, in the Philippines, serious outbreaks of surra with high mortality rates have occurred in horses, buffaloes, cattle, small ruminants, and pigs [94, 102, 106, 127].

The financial losses due to surra are high, but treatment is cost effective. Recent estimates using a bioeconomic infectious disease model suggest that a typical village in the Philippines with livestock (80 buffaloes, 40 cattle, 200 pigs, 150 goats/sheep, and 15 horses), affected with moderate to severe surra, can lose as much as US $158,000 every year. However, it was demonstrated that the same village could earn the same amount of money if treatment was used [128]. The model was developed using a vast amount of data from a 4-year field survey in Mindanao, in the Philippines, where surra is highly endemic. By comparison, the previous estimate of losses due to surra in the Philippines was only US $0.1 million yearly nationwide [102]. The previous estimate was only based on the limited mortality data submitted to the government [102], whilst the current data were based on losses due to mortality, low reproduction, diagnosis, treatment costs, and replacement costs [128]. However, the present monetary estimates of losses due to surra may still be an underestimation because some factors were not taken into account: losses from weight loss, carcass quality, milk production, draught output, and reduction in selling price. In Mindanao, the market price for *T. evansi*-infected animals is very low (30–50% lower). The high financial losses caused by *T. evansi* infection in livestock in endemic areas have a great social impact on poor farmers and their families who are dependent on their livestock for farm activities and income. The need to import replacement stock from other sources is also an additional financial burden for marginal low-income farmers.

Financial losses due to surra can be avoided by adopting an effective control approach that includes an effective control strategy. In surra-endemic areas in Pantanal, Brazil, where the cattle industry is significant and horses are used for herding livestock, year-long monitoring and treatment of horses with diminazene aceturate have been shown to be the most economical treatment option, with a total net benefit of more than US $2 million per year [116]. Nevertheless, this strategy assumed that the drug is 100% effective against *T. evansi,* which is unlikely to be the case, particularly in areas where drug resistance exists. Similarly, in the Philippines, targeted treatment of all animals infected with surra throughout the year using a highly effective drug (e.g., melarsomine dihydrochloride) is the most beneficial treatment strategy. Biannual mass treatment of all livestock species in a village is also financially viable but may result in drug resistance amongst *T. evansi* isolates [128].

As a conclusion on the epidemiology, impact, and control of surra, a regular and sustained effective surveillance system must be carried out to monitor and assess the efficacy of treatment strategy, and support the control efforts. The success of any surveillance and control activities for surra is depending, amongst others, on (i) financial and resources allocation, (ii) support from the stakeholders (including local government officials), (iii) commitment of the surveillance and technical staff, and (iv) effective reporting system and close cooperation with the farmers. Animal owners should be properly educated on surra (e.g., impact, biology, and clinical signs), be empowered in monitoring their animals for any evidence of the disease, and be aware on whom and how to report to concerned authorities for confirmation and treatment. Regular monitoring and immediate treatment of animals with surra with an effective trypanocide were shown to be economically beneficial [128]. Random sampling of livestock using a combination of appropriate diagnostic tests (serological and parasitological or molecular) must also be regularly carried out in endemic locations to assess efficacy of the control program and detect potential asymptomatic carriers. Subclinical surra may occur in healthy animals (e.g., buffaloes, cattle) [106, 129]); they are a real infection threat (as potential sources of the parasite) to other animals, including highly susceptible ones such as horses, camels, and dogs [130, 131]. Therefore, identification and subsequent treatment of subclinically infected livestock are significant to any efforts to control surra amongst livestock.

## 6. Zoonotic Aspects

*Trypanosoma evansi* is morphologically indistinguishable from the bloodstream form of *Trypanosoma brucei *spp., the causative agents of human sleeping sickness (African Human Trypanosomosis, HAT), that is, *T. b. rhodesiense* and *T. b. gambiense* and the pathogen of animal Nagana, *T. b. brucei*. However, the host range of *T. evansi* is restricted to nonhuman animals because of its susceptibility to cytolysis by the trypanolytic factor in normal human serum (NHS). The trypanolytic factor was first found as a consistent component of high density lipoprotein. A later subfractional study showed that this component was apolipoprotein L-1 (ApoL-1) [132, 133]. The main components involved in NHS-mediated trypanolysis are the primate-specific apolipoprotein L-I (apoL1) and haptoglobin-related protein (Hpr), which are associated with a minor subfraction of HDLs and an IgM/apolipoprotein A-I (apoA1) complex, respectively, termed trypanosome lytic factor (TLF) 1 and TLF2. The TLF1-Hpr-haemoglobin (Hb) complex binds to the trypanosome haptoglobin (Hp)-Hb receptor, which triggers efficient uptake of TLF1 and subsequent trypanosome lysis [134]. The trypanolytic activity of ApoL-1 is caused by the formation of an ionic pore in an acid pH environment [135]. This requires the translocation of the molecular membrane into a lysosome membrane, possibly by the haptoglobin-hemoglobin (Hp-Hb) receptor [136]. Apo L-1 are human apolipoproteins considered as the trypanolytic factor present in NHS. They provide innate protection to humans from infection by African trypanosomes, such as *T. evansi*, *T. b. brucei,* and others, with the exception of *T. brucei rhodesiense *and *T. b. gambiense,* which developed resistance mechanisms [137]. Thus,* T. evansi* has long been considered a nonhuman infective species similar to *T. b. brucei*. However, in 2005, a human case of trypanosomosis caused by *T. evansi* was reported in a farmer from the Chandrapur district in the Maharashtra State, India [138–140].

In this case, the man had fluctuating trypanosome parasitaemia associated with febrile episodes for several months. In the absence of central nervous system invasion, the patient has been treated successfully with suramin. Contamination by contact of a wound with infected animal blood was suspected [140].

The infection was puzzling because the trypanosomes isolated from the patient were found to be typical *T. evansi* based on the analysis using molecular biology [141]. However, later it was demonstrated that the infection was due to the frameshift mutations in both Apo L-1 alleles in the patient [142]. This led to an unexpected termination of protein translation by internal stop codons [142], which resulted in a total absence of Apo L-1. Without Apo L-1, the patient lost his protection against *T. evansi* and the infection thus developed human surra [140]. An investigation is urgently required on the distribution of mutated Apo L-1 alleles in the populations and the exposure of the population to *T. evansi* in prevalent areas in order to determine the potential of *T. evansi* to infect humans.

A serologic screening was carried out in the surrounding area near the first human case. Serum or blood from 1,806 people from the patient's village of origin was tested with the CATT/*T. evansi*. The results showed that 4.5 to 22.7% were positive samples (with serum or blood, resp.). No trypanosome was detected in the blood of 60 people that were highly positive. These results suggest that the human population is frequently exposed to* T. evansi* [143]. The specificity of the CATT has not been investigated in humans in Asia. Thus, further research is required.

Given the wide distribution of this parasite in developing countries, a large part of the population is at risk from infection, either by direct contact (percutaneous infection), or per-oral or, more likely, via bites from blood-sucking insects that have previously fed on infected animals. Thus, although there are no reports on the prevalence of mutated Apo L-1 alleles in the populations, people are still at risk, particularly immunosuppressed individuals living in the regions where *T. evansi* is endemic.

In fact, there were some suspected cases of human trypanosomosis caused by *T. evansi*. The earliest case was reported by Gill [2], in a scientist infected while pipetting infected blood. The symptoms were insomnia, tachycardia, enlargement of liver, spleen, and lymph nodes, and loss of recent memory. In this case, nervous invasion was likely and was successfully treated with atoxyl (*p*-aminophenylarsenic acid). More recently, a case was reported on ProMED-mail in 1999 [144]. The case was not officially published, but trypanosomes isolated from the patient were confirmed as *T. evansi* using PCR (W. Gibson, University of Bristol, UK, personal communication). A further four cases of suspected human trypanosomosis caused by *T. evansi* infection were reported in India with one mortality [145, 146]. However, no confirmatory reports have been obtained until now. Currently, a human infection by *T. evansi* was also reported from Egypt although no details were provided regarding the status of the gene of Apo L-1 in the patient [147].

The resistance to ApoL-1 of pathogens of African sleeping sickness was demonstrated by at least two different strategies with the neutralization by SRA in the case of *T. b. rhodesiense* [148] or the limited sublethal uptake of ApoL-1 in the case of *T. b. gambiense* [149]. Actually, it was recently demonstrated that the loss of the Hp-Hb receptor reduced the susceptibility of trypanosomes to TLF-1, and to a lower extent to TLF-2, suggesting that both toxins can be taken up via the Hp-Hb receptor, but those alternative pathways exist [150]. Although SRA is absent in *T. evansi*, a pseudo gene called SRABC has been confirmed [151]. Given the fact that *T. evansi* is directly transmitted by blood sucking insects with no development stages (life cycle) in the vector (like those found in *T. brucei*), the horizontal gene transfer of SRA observed in *T. b. rhodesiense* is highly unlikely. However, tolerance to NHS among the *T. evansi* stocks was reported [152]. Unfortunately, it was noted that the tolerance to these stocks could be enhanced by continued exposure to NHS [152]. Similar results were found in *T. b. brucei*, genetically very close to *T. evansi*, after 9 months of *in vivo* selection with NHS [153]. It was suggested that the reduction of ApoL-1 uptake might be associated with the low level of haptoglobin-hemoglobin receptor expression [154]. Whether *T. evansi* could develop any of the above strategies or others to resist ApoL-1 lysis remains uncertain. The lack of innate protection of ApoL-1 in humans or the development of new capacities in parasites to counter innate immune responses could lead to the evolution of a major new trypanosome pathogen.

Lastly, although *T. evansi* is still not considered to be a zoonosis, it is wise to remain cautious. The same applies to other trypanosomes, such as *Trypanosoma lewisi*, which as yet is considered to be atypical in humans [155].

In addition to these cases, other reports of cases where humans are infected by *Trypanosoma lewisi* and *T. evansi* [156–158] have led to the creation of a new network to coordinate information and research on atypical human infections caused by animal trypanosomes (NAHIAT). (The NAHIAT, Network on Atypical Human Infection by Animal Trypanosomes, was created in May 2011. It is coordinated by the Institute of Research for Development (IRD) and the Center for International Collaboration on Agricultural Research for Development (CIRAD) with the support of FAO, OIE, WHO, and a number of international research institutes and universities. Contacts: Dr. Philippe Truc <philippe.truc@ird.fr> and Dr. Marc Desquesnes <marc.desquesnes@cirad.fr>.) In this new context, further cases of human infections by *T. evansi *have already been reported [159].

## 7. Conclusions and Perspectives

The exact origin of *T. evansi* has not been fully clarified. In fact, in reality there may be “several” *T. evansi* [160]. Nonetheless, most of the parasite's dominant properties are well known, with the exception of its particular ability to induce immunosuppressive effects. This aspect requires additional investigation, which in turn could provide the opportunity to further our understanding of infectious parasitic immunosuppression.

This brief review of the fundamental knowledge of *T. evansi* has provided the opportunity to emphasise the fact that this parasite has an unlimited geographical distribution. Its distribution is directly related to its almost unlimited range of hosts. The same applies to its unlimited range of potential reservoirs and its unlimited, nonspecific, and ubiquitous range of potential vectors. Lastly, the loss of some DNA material has made *T. evansi* a better parasite, inasmuch as it is less specific in terms of vector and geographical distribution! By losing its dependency on tsetse flies, which are ecologically restricted to a specific area in Africa, *T. evansi* has travelled unlimited distances and found vicariant hosts and the necessary reservoirs and vectors for its successful expansion. It is important to emphasise that the geographical expansion of *T. evansi* is not limited, and recent outbreaks of surra in Spain and France are of serious concern for the health authorities. It is important to note that in Europe and Australia, this restless parasite should be monitored closely! Similarly, the undocumented situation in Turkey, Bulgaria, and neighbouring countries, such as Greece, should be given more attention. Surra might well become established unseen in less susceptible domestic and wild fauna in Europe, before an outbreak is reported with fatal cases among the more susceptible domestic hosts. Sanitary measures should be improved because, as the recent outbreaks in Spain and France have shown, healthy or inapparent carriers of *T. evansi* can be exported from infected to noninfected areas of the world. The necessary measures are proposed in this paper.

The main vectors have been identified as Tabanids and *Stomoxys*. However, theoretically the vectors are unlimited and a number of questions have yet to be answered. What is the relative role of *Haematobia* sp., which is sometimes very abundant in some hosts, such as camels and buffaloes? An even more important question is the following: what is the potential of *Stomoxys* sp. for delayed transmission? It could play a role in interherd transmission, including the link between domestic and wild animals. If this was the case, it would determine the framework for controlling the disease in situations where domestic animals are found in the same areas as other domestic or wild hosts that potentially act as a reservoir for the parasite. Determining the capacity of *Haematobia* and *Stomoxys* to transmit *T. evansi,* as well as all the other mechanically transmitted pathogens (bacteria, viruses, and parasites), at intervals of a few hours or days is a major challenge for research. The results would influence risk evaluation and means of controlling the parasite, especially in the case of new disease emergence in countries previously free of infection. Other modes of transmission should also be investigated, such as leeches, ticks, and bugs that may even act as passive carriers.

It is still unclear why *T. evansi* is considered to be non-pathogenic to cattle in Africa and Latin America and yet is a major pathogen in Asia. The answer may be found in the genetic make-up of the parasites and/or the cattle themselves. However, the variability of the effects of surra infection in a host from the “same” species is a general feature. Highly variable effects have been recorded in cattle, as well as buffalo, sheep and even horses and dogs. Infected animals may die in over 90% of cases or appear perfectly healthy in highly enzootic conditions. In the field, these variations may well be related to a complex that includes host and parasite genetics, epidemiological situation, vector pressure, control measures, and other sanitary or zootechnical parameters, which could be seasonal. However, we still do not fully understand the pathogenicity of *T. evansi* in Asian cattle and buffalo. It remains one of the greatest mysteries of this amazing parasite.

However, research in Indonesia and the Philippines has confirmed that South East Asian isolates of *T. evansi* are more pathogenic than African isolates and that isolates of *T. evansi* from the Philippines are more pathogenic than those collected in Indonesia [161, 162]. These results may partly explain why surra is a much more severe disease in the Philippines compared to other endemic countries [102, 106, 107, 128]. Our ultimate objective is to help focus control programmes on high-risk areas by identifying the presence of these “pathogenic” strains using PCR. Research on the proteomic characterisation of *T. evansi* is aimed at identifying proteins involved in this pathogenic process.

The basic knowledge on *T. evansi *suggests that there is renewed interest in the parasite, which is spreading and has an economically important impact. This review has stressed the highly variable characteristics exhibited by a taxon called *Trypanosoma evansi* under various circumstances, in various geographical locations, and in interactions with specifically and genetically varied hosts, reservoirs, and vectors. The large range of interactive phenotypic aspects probably reveals a genetic diversity that should be evaluated via complementary studies and reviews on the molecular epidemiology of *Trypanosoma evansi* in order to help further our understanding of the parasite's polymorphic features.
